# Changes in equity of maternal, newborn, and child health care practices in 115 districts of rural Ethiopia: implications for the health extension program

**DOI:** 10.1186/s12884-015-0668-z

**Published:** 2015-10-05

**Authors:** Ali Mehryar Karim, Addis Tamire, Araya Abrha Medhanyie, Wuleta Betemariam

**Affiliations:** The Last Ten Kilometers Project, JSI Research & Training Institute, Inc., PO Box 13898, Addis Ababa, Ethiopia; Ministry of Health, Federal Democratic Republic of Ethiopia, PO Box 1234 Addis Ababa, Ethiopia; Department of Public Health, College of Health Sciences, Mekelle University, Mekelle, Ethiopia

**Keywords:** Health extension program, Maternal, Newborn and child health, Universal health coverage, Equity

## Abstract

**Background:**

Reducing within-country inequities in the coverage of maternal, newborn, and child health (MNCH) interventions is essential to improving a country’s maternal and child health and survival rates. The community-based health extension program (HEP) of Ethiopia, launched in 2003, aims to provide equitable primary health care services. Since 2008 the Last Ten Kilometers Project (L10K) has been supporting the HEP in promoting equitable MNCH interventions in 115 districts covering about 14 million people. We report the inequities in MNCH programmatic indicators in 2008 and in 2010 in the L10K areas, along with changes in equity between the two survey periods, and the implications of these results for the national program.

**Methods:**

The study used cross-sectional surveys of 3932 and 3867 women from 129 representative *kebeles* (communities) conducted in December 2008 and December 2010, respectively. Nineteen HEP outreach activity coverage and MNCH care practice indicators were calculated for each survey period, stratified by the inequity factors considered (i.e. age, education, wealth and distance from the nearest health facility). We calculated relative inequities using concentration indices for each of the indicators and inequity factors. Ninety-five percent confidence intervals and survey design adjusted Wald’s statistics were used to assess differentials in equity.

**Results:**

Education and age related inequities in the MNCH indicators were the most prominent (observed for 13 of the 19 outcomes analyzed), followed in order by wealth inequity (observed for eight indicators), and inequity due to distance from the nearest health facility (observed for seven indicators). Age inequities in six of the indicators increased between 2008 and 2010; nevertheless, there was no consistent pattern of changes in inequities during that period. Some related issues such as inequities due to wealth in household visits by the health extension workers and prevalence of modern family household; and inequities due to education in household visits by community health promoters showed improvement.

**Conclusions:**

Addressing these inequities in MNCH interventions by age, education and wealth will contribute significantly toward achieving Ethiopia’s maternal health targets for the Millennium Development Goals and beyond. HEP will require more innovative strategies to achieve equitable MNCH services and outcomes and to routinely monitor the effectiveness of those strategies.

**Electronic supplementary material:**

The online version of this article (doi:10.1186/s12884-015-0668-z) contains supplementary material, which is available to authorized users.

## Background

Addressing inequities in maternal, newborn and child health (MNCH) is a key strategy to improve maternal, newborn and child health and survival, for the achievement of Millennium Development Goals (MDGs) 4 and 5 (reducing child mortality and improving maternal health, respectively) that are set for 2015 [1], and for further improving MNCH beyond the MDGs. The Government of Ethiopia, recognizing the need to provide its people with equitable access to promotive, preventive and selected curative health services, launched the health extension program (HEP) in 2003. The HEP—a community—or *kebele-*based health service delivery system—is the backbone of Ethiopia’s plan to achieve its priorities in the health sector, including the country’s health-related MDGs. (A *kebele* is the smallest administrative unit of the country, with a population of about 5000.) The main strategies of the HEP include establishing a health post and training and deploying two government-salaried female health extension workers (HEWs) in every *kebele* of the country. The HEP provides a package of services with 16 components in four major program areas: family health services, disease prevention and control, hygiene and environmental sanitation, and education and communication [2–4]. Health centers, staffed with nurses, midwives and health officers, provide administrative, logistical, technical, and referral support to the HEWs. The health centers provide a wide range of mainly curative services and are being equipped to provide basic emergency obstetric and neonatal care. One health center provides support to five health posts and forms the primary health care unit, which is linked with a primary hospital to provide more specialized services including comprehensive emergency obstetric and newborn care [5].

The HEP has achieved universal coverage by establishing at least one health post and deploying at least two HEWs in nearly all of the 15,000 *kebeles* in Ethiopia. The number of health centers supporting the health posts increased from about 800 in 2005 to more than 2000 in 2011 [6]. At first, the HEWs spent 75 % of their time conducting household visits and community outreach activities, training families to adopt desirable health behaviors and practices and to serve as “model families” in their neighborhood, and organizing communities to participate in the expansion of HEP services. Families are taught hygiene, environmental sanitation, family planning, MNCH, disease prevention and control practices. Families that adopted 75 % of the healthy practices are said to ‘graduate’ as a ‘model family’ household [7]. Community health volunteers referred as community health promoters (CHPs), who are from model family households and willing to volunteer have been supporting the HEWs in providing HEP services, with a density of one CHP for every 25 to 30 households [2–4]. The HEWs and CHPs used a family health card (FHC), a booklet with pictorial messages, to promote focused MNCH care behaviors and practices.

Recently the government’s Ministry of Health implemented a new policy to increase the density of CHPs to one for every five households and to rename the CHPs as health development army members [7]. With the initiation of integrated community case management of common childhood illnesses in 2011, the HEWs now spend about 50 % of their time at health posts [8].

Since December 2008, the Bill & Melinda Gates Foundation has funded activities of the Last Ten Kilometers Program (L10K) implemented by JSI Research & Training Institute, Inc., to support the HEP to improve MNCH outcomes in 115 rural districts (*woredas*), i.e., about three thousand *kebeles*, of four regions of Ethiopia—Amhara, Oromia, Tigray, and Southern Nations, Nationalities and People’s—thus contributing toward the country’s achievement of MDGs 4 and 5. To do so, L10K project works to ensure that interactions between HEP front-line health workers—i.e., HEWs and CHPs (currently the health development army members)—and households to provide MNCH services will be more frequent, of higher quality, more cost-effective, and more equitable [9].

The Ethiopia Demographic and Health Survey (EDHS) 2011 indicated that maternal and child health indicators have improved since the introduction of the HEP. Between 2005 and 2011, the contraceptive prevalence rate increased from 15 to 29 %, unmet need for family planning declined from 34 to 25 %, pre-natal care coverage increased from 28 to 44 %, deliveries assisted by skilled providers increased from six to 10 %, institutional deliveries increased from four to 10 %, births protected from neonatal tetanus increased from 32 to 48 %, and measles vaccination coverage increased from 29 to 56 %, while mortality in infancy and under age 5 declined from 77 and 123 deaths per 1000 live births, respectively, to 59 and 88 deaths per 1000 live births [10]. Similarly, the L10K baseline and follow-up surveys (conducted in December 2008 and December 2010) conducted in the 115 L10K districts documented significant improvements in MNCH care behavior and practices [9].

In order to provide universal primary health care, all services provided by the HEWs (and CHPs) are free of charge. The epicenter of the primary health care unit, i.e., the health centers, has user fees, but either MNCH services are provided free to all persons or user fees for these services are waived for the poor [4]. It is critical to monitor whether the HEP has been able to establish an equitable health system to achieve universal primary health coverage. Culyer [11] defines an equitable health system as one that “treats those with equal need equally and those in greater need … in proportion to that greater need”.

Traditionally, equity in global health has been measured by observing the differences in health care practices according to household wealth [1], mainly because improving the health of the poor has been the top priority among international development agencies [12–14]. However, according to the Culyer definition, equity in health can also have other dimensions such as age, education or residence (i.e., geographical location or distance from a health care provider). For instance, young women are at higher risk for adverse consequences of childbearing than women in their thirties, and children of young women are also at higher risk for morbidity and mortality and thus in greater need of MNCH services [15–18]. Similarly, women with low levels of education also have greater need for MNCH services because they are at a higher risk of maternal morbidity and mortality, and the children of relatively uneducated mothers are likewise at higher risk of adverse health outcomes [19, 20]. Furthermore, one HEP strategy for achieving universal coverage of primary health care has been to reduce the distance to service delivery points.

The community-based strategies of the HEP and L10K that influence universal and equitable accesses to priority MNCH services are listed in Table 1. Nevertheless, there are no studies to assess the adequacy of the HEP and L10K’s efforts to do so. Thus, this study uses data from the L10K surveys to assess the equity of use of MNCH services in L10K areas and whether there was any change in equity between the baseline and follow-up surveys. Equity of MNCH outcomes was examined along four dimensions: women’s age, education, wealth, and household distance from the nearest health facility.

**Table 1:** Community-based strategies implemented during the study contextual period that were aimed at establishing an equitable health system

Health extension program	The Last Ten Kilometers Project
- Establish one health post with two HEWs for every 5,000 populations - Free of charge - Health education during interaction with clients at health posts, communities and households - Train model families to adopt healthy behaviors and practices that influence their neighbor to do the same - Organize CHPs to promote HEP services - Community mobilization	- Provide refresher training to HEWs on maternal and newborn health - Train HEWs to organize CHPs to identify the target population for MNCH to promote and ensure HEP services, provide health messages to the target populations in hard to reach areas - Conduct review meetings with the HEWs at the *woreda*-level to exchange best practices - Provide supportive supervision visits to the HEWs to reinforce their skills and address performance gaps

## Methods

### Study design

This study is a secondary analysis of two cross-sectional surveys conducted in December 2008 (the L10K baseline survey) and December 2010 (the L10K follow-up survey) to assess the equitability of the MNCH interventions in L10K areas. Results of the two surveys were compared to determine whether there have been any changes in the inequities between the two survey periods.

### Data collection

The survey collected information from three target groups of women. Specifically, family planning information was collected from women age 15 to 49 years; maternal, newborn, and infant health and nutrition information from women with children in their first year of life; and child immunization and childhood illness information from women with children age 12 to 23 months. The survey instruments for the three target groups were adapted from Demographic and Health Survey [10] and Saving Newborn Lives questionnaires. The latter was obtained through personal communication from Saving Newborn Lives Program in Ethiopia. The questionnaires were translated into the three major local languages (Amharic, Oromifa, and Tigrigna). In the Southern Nations and Nationalities People’s Region, which has 11 additional languages, the interviewers translated from Amharic while administering the questionnaires. Verbal consent was sought and documented by the interviewer. If the respondent was under 18 years old, then consent was sought from her husband, parents or guardian. As it was expected that most of the respondents could not read or write, written consent was not sought. Ethical clearance was obtained from the Ethiopian Public Health Association.

The details of sample-size estimation during the baseline and follow-up surveys are provided elsewhere [9]. For the purpose of the present study, the study sample was restricted to the 129 *kebeles* that were visited during baseline survey and during follow-up survey to interview the target respondents. The baseline survey sample included 3932 women respondents; among them, 2580 women were target for collecting family planning information, 1548 women had children in their first year of life, and 1290 women had children 12 to 23 months old. The follow-up survey sample included 3867 respondents, among whom about 1548 women were from each of the three target groups. The smallest sample size (i.e., 1290 women with children 12 to 23 months) was adequate to measure a point prevalence of 50 % with ±4 percentage-points precision when the cluster design effect was set at 2.0 and two tailed alpha error was set at 0.05.

Two-stage stratified cluster sampling was used to select households. At the first stage, *kebeles* were selected as clusters with probability proportional to their estimated population sizes, stratified by region At the second stage, the 30 by seven cluster survey strategy, which is commonly used for monitoring the coverage of childhood immunization services, was adapted to obtain information from the three target groups [21]. In brief, the first household was selected from the middle of the *kebele* and then every fifth household was visited, moving away from the center, and all women in that household were interviewed if they were within the target population and if they gave consent. Thus, if women age 15–49 years had a child between 0 to 11 months of age she was interviewed for the family planning questionnaire as well as the questionnaire for women with children 0 to 11 months. From each *kebele*, a quota of 20 interviews with women age 15–49, 12 with women who had children zero to 11 months old, and ten women with children 12–23 months was set during the baseline survey, and a quota of 12 respondents from each of the three target groups was set for the follow-up survey. After reaching the quota for women age 15–49 in a *kebele*, the interviewers sought only to conduct interviews for the other target groups.

The interviewers and supervisors were health professionals from health centers and woreda health offices who were knowledgeable of the services provided by the HEP., and they received five days of training including a day of field practice. They did not interview in the areas under their supervision. Survey supervisors and regional coordinators were trained to monitor and supervise the work and ensure data quality. Regional coordinators were consultants hired to monitor the quality of data collection by revisiting randomly selected households to validate responses. The total time required to complete each round of surveys, including the training period, was about a month. Data were entered twice and discrepancies were resolved with reference to the original forms.

### Measurement and statistical analysis

Nineteen MNCH indicators were the outcomes of interest. They were grouped into two broad categories: (1) HEP outreach activity coverage indicators, which were measured among unique respondents from all three target groups of women; and (2) MNCH care practice indicators. MNCH indicators were sub-categorized into (i) family planning, which was measured among the respondents of reproductive age; (ii) maternal and newborn health, which included care behavior and practices during the most recent pregnancy as reported by women with children in their first year of life; and (iii) child health, which was measured either among all women with children age 23 months or less or among women with children age 12 to 23 months, as appropriate for each question. The definitions of the indicators are given in Table 2.

**Table 2:** Definition of HEP outreach activity coverage and MNCH care practice indicators

MNCH Indicator	Definition
(1) HEP outreach activity coverage	
Households visited by HEWs	The percentage of respondents whose households were visited by HEWs to discuss about health related issues within six months prior to the survey
Households visited by CHPs	The percentage of respondents whose households were visited by CHPs to discuss about health related issues within six months prior to the survey
Model family households	The percentage of the respondents whose household graduated as a model family or working towards it
Households with family health card	The percentage of respondents whose households have family health card. FHCs are distributed by HEWs to all women of reproductive age in a household. The cards are used as a tool to provide health education for promoting MNCH; as such all households with women of reproductive age should possess the card
(2) MNCH care practices	
(i) Family planning	
CPR among women (married or in union)	The percentage of women and/or their partners who were using any method of contraception to delay or avoid getting pregnant during the survey
(ii) Maternal and newborn health	
Received any ANC from health facility	The percentage of women who went to a health facility for antenatal care during last pregnancy
Received iron supplementation	The percentage of women who took iron supplementation during last pregnancy
Received TT2+	The percentage of women who received two or more tetanus toxoid injections (TT2+) during last pregnancy
Took any birth preparedness measure	The percentage of women who took any preparation for delivery (such as financial, transportation) when they were last pregnant
Delivery at health facility	The percentage of women who gave their last childbirth at a health facility
Delivery assisted by skilled attendant	The percentage of women who were assisted by a health professional (doctor, nurse or midwife) during last childbirth
Received any PNC	The percentage of women who were visited by HEWs or CHPs at home for any PNC or newborn care within 40 days of last childbirth
Took thermal care of their newborn	The percent of women who dried and wrapped their last newborn immediately after birth, delayed bathing the newborn by six hours or more, and always maintained skin-to-skin contact with the baby
Took clean cord care of their newborn	The percent who cut the umbilical cord of their last newborn with a sterile instrument, tied the cut end of the cord with sterile thread, and applied nothing to the cut end of the umbilical cord
Gave baby first milk to their newborn	The percentage of women who gave first milk (colostrums) to their last newborn
(iii) Child health	
Households contacted by HEWs for child health and nutrition	The percentage of women with children zero to 23 months who were visited by any health worker (i.e., HEW or CHP or both) to provide advice on child health and nutrition
Children with ARI received any treatment	The percentage of children between 0 and 23 months who had an episode of acute respiratory infection (ARI) during the two weeks preceding the survey were taken to any health provider
Children with diarrhea received ORT	The percentage of children between 0 and 23 months who had an episode of diarrhea during the two weeks preceding the survey who were given oral rehydration therapy (ORT)
Children received all vaccines	The percentage of children between 12 and 23 months who received all childhood vaccines

Inequities in the 19 MNCH indicators and changes in inequities between the two surveys were investigated and analyzed by age, education, wealth and distance from the nearest health facilities. The wealth index score, with a mean of 0 and a standard deviation of 1, was constructed for each household using principal component analysis of 12 household possessions (electricity, watch, radio, television, mobile phone, telephone, refrigerator, table, chair, bed, electric stove, and kerosene lamp) and two household characteristics (type of latrine and water source) [22]. Households were then ranked according to their wealth score and divided into three terciles indicating the poorest, medium, and least poor households. This ranking was done separately for the baseline and follow-up surveys. The wealth index score has been shown to be a reasonable proxy for estimating household wealth status in the absence of income or consumption data [23–26]. Distance was defined as the time required to travel to the nearest health facility (health post, health center or any other) using the most common mode of transport.

The presence of inequities in an indicator of interest across wealth status was assessed using the wealth inequity concentration index with its standard errors, adjusted for sampling weights and cluster survey design effects. Wald’s statistics, adjusted for survey design, were also used to assess differentials in an indicator according to the wealth terciles at the baseline survey and at the follow-up survey. The variance estimates for the Wald statistics was obtained using Taylor series linearization method implemented by [27, 28]. Inequity was defined as pro-rich or bottom (B) if the indicator of interest was the lowest in the most disadvantaged group (the poorest tercile) and defined as pro-poor or top (T) when it was the opposite.

Whether there were changes in the differentials of indicators by wealth terciles between the survey periods were determined by testing the interaction terms between survey periods and wealth tercile indicator variables using a logit model. Wald’s statistics was used for the purpose, adjusted for sampling weights and cluster survey design effect.

Similarly, concentration indices for the other three inequity factors (age, education, and distance from a health facility) were constructed, and Wald’s statistics were also applied to assess inequities and whether they had changed between the survey periods. It is likely that the distribution of the health outcomes of interest across wealth status may be confounded by educational or other socio-demographic characteristics of the respondent. As such, the concentration index was standardized using indirect methods [29]. For instance, the wealth inequity concentration index was standardized for the confounding variables of respondent’s age, education, and household distance from the nearest health facility.

The concentration index can have values that range between +1 and –1. The wealth inequity concentration index has a negative value if the outcome is more favorable for the poor and a positive value if the outcome is more favorable for the rich. If the value of the index was significantly (*p* < 0.05) different from zero, then it was concluded that wealth inequity for the indicator was statistically significant. Similarly, a statistically significant negative value for the age inequity concentration index indicated that the outcome of interest was more favorable among younger women, whereas a statistically significant positive value indicated that the indicator was higher among older women. A statistically significant negative value for the education inequity concentration index indicated that the indicator was higher among less educated women and a statistically significant positive value indicated that it was higher among more educated women. With regard to the inequity concentration index on distance (operationalized as travel time) from a health facility, the distance was divided by –1, so that a negative value for the index indicated that the outcome of interest was concentrated among women who lived further away from a health facility, whereas a positive value indicated that the indicator was concentrated among those women who lived closer to a health facility.

Statistically significant changes in MNCH indicators between the two survey periods were assessed using Pearson’s chi-squared statistics, adjusted for survey design. Statistically significant changes in concentration indices were assessed using *t*-tests with linearized standard errors adjusted for survey design. The two-sided alpha error was set at five percent for the bivariate analyses.

## Results

### Sample characteristics

The distributions of the L10K baseline and follow-up survey respondents by region, age, and parity were similar between the two surveys (Table 3). Although the populations of the two surveys were statistically significantly different (*p* < 0.05) in terms of marital status and education, the differences were small (i.e., less than 2 percentage-points).

**Table 3:** Background characteristics of survey respondents, baseline (December 2008) and follow-up (December 2010) surveys

Background variable	Category	Baseline (%)	Follow-up (%)	
No. of respondents		3,932	3,867	
Region	Tigray	13.6	14.5	
	Amhara	41.1	41.6	
	Oromia	26.2	25.1	
	SNNP	19.1	18.8	
Age group	15-19	7.1	6.9	
	20-34	72.3	71.5	
	35-49	20.7	21.6	
Marital status	Not married	6.5	7.9	**
	Married	93.5	92.1	
Education	None	82.1	80.2	**
	Primary	12.1	12.3	
	Higher	5.9	7.5	
Number of children	0	2.7	2.9	
	1	16.5	17.3	
	2	15.9	16.5	
	3	15.2	16.1	
	4+	49.7	47.3	
Religion	Orthodox	61.8	64.4	
	Protestant	13.0	12.3	
	Muslim	24.1	22.5	
	Other	1.2	0.9	
Wealth terciles	Poorest	36.1	36.7	
	Medium	38.0	36.3	
	Least poor	25.9	27.0	
Distance to any health facility	<30 min.	54.2	62.9	**
	30 min - <1 hr	23.6	27.7	
	1+ hr	22.2	9.4	

**p < 0.05

There has been significant change in access to a health facility in the L10K areas. Between the two survey periods, the proportion of the respondents whose household was within 30 min of any health facility increased from 54 % to 63 %, while the proportion of women who were an hour or more away from any health facility declined from 22 % to 9 %.

### Improvements in access and utilization of MNCH services

Between baseline and follow-up surveys there were statistically significant (*p* < 0.05) improvements in 17 of the 19 MNCH indicators of interest analyzed (Table 4). Only the changes observed in percentages of children with acute respiratory infection (ARI) who received any treatment and of children with diarrhea who received oral rehydration therapy (ORT) were statistically not significant (*p* > 0.05).

**Table 4:** Change in HEP outreach activity coverage and MNCH care practice indicators between baseline (December 2008) and follow-up (December 2010) surveys

Indicators	Baseline (%)	Follow up (%)	Change (%)	
HEP outreach activity coverage	*N* = 3,932	*N* = 3,867		
1. Households (HHs) visited by HEWs	36.6	47.2	10.5	**
2. HHs visited by CHPs	15.9	30.7	14.8	**
3. Model family households	9.4	28.0	18.6	**
4. HHs with family health card	5.3	32.4	27.1	**
MNCH care practices				
(1) Family planning	*N* = 2,580	*N* = 1,548		
5. CPR among married or in union women	26.9	37.4	10.6	**
(ii) Maternal and newborn health (M&NH)	*N* = 1,548	*N* = 1,548		
6. Received any ANC from health facility	50.8	66.5	15.8	**
7. Received iron supplementation	10.4	27.8	17.3	**
8. Received TT2+	40.4	42.4	2.0	**
9. Took any birth preparedness measure	68.8	74.1	5.4	**
10. Delivery at health facility	5.2	10.6	5.4	**
11. Delivery assisted by skilled attendant	9.5	14.6	5.1	**
12. Received any PNC	4.2	14.0	9.8	**
13. Took thermal care of their newborn	11.6	23.1	11.5	**
14. Took clean cord care of their newborn	31.1	38.4	7.2	**
15. Gave baby first milk to their newborn	44.6	51.4	6.8	**
(iii) Child health	*N* = 2,838	*N* = 3,084		
16. HHs contacted by HEWs for child health and nutrition	11.0	23.9	12.9	**
17. Children with ARI received treatment	31.3	35.3	4.0	
18. Children with diarrhea received ORT	43.8	48.3	4.6	
19. Children received all vaccines	44.3	50.8	6.4	**

**p < 0.05

### Inequities in HEP outreach activity coverage and MNCH care practice indicators

Inequities (*p* < 0.05) in HEP outreach activity coverage and MNCH indicators according to age, education, wealth and distance from the nearest health facility during the follow-up survey, along with changes (*p* < 0.1) in the inequities between baseline and follow-up surveys using Wald’s statistics and the concentration indices, are presented in Additional file 1: Table S1 and summarized in Table 5. In the follow-up survey there were bottom inequities for age and education for 13 of the 19 indicators, bottom inequities for wealth for eight indicators, and bottom inequities for distance from the nearest health facility for seven indicators. Results using Wald’s statistics and concentration indices corroborated for only 19 of 41 statistically significant bottom inequities observed during the follow-up survey period.

**Table 5:** Inequities in the HEP outreach activity coverage and MNCH care practice indicators according to age, education, wealth, and distance to any health facility during follow-up survey and the changes in inequities in those indicators between baseline and follow-up surveys based on Wald’s statistics and concentration indices (CI)

Indicators	Age inequity				Education inequity				Wealth inequity				Inequity due to distance to any health facility			
	Follow-up		Change		Follow-up		Change		Follow-up		Change		Follow-up		Change	
	Wald	CI	Wald	CI	Wald	CI	Wald	CI	Wald	CI	Wald	CI	Wald	CI	Wald	CI
HEP outreach activity coverage																
1. Households visited by HEWs	B	B	N	N	B	B	N	N	N	N	↓	N	B	B	N	N
2. HHs visited by CHPs	B	B	N	N	N	B	↓	N	N	N	N	N	N	N	N	N
3. Model family households	B	B	N	N	N	N	N	N	N	N	↓	N	N	N	N	N
4. Households possessing a family health card	B	B	N	N	B	B	N	N	N	N	↑	N	N	N	N	N
MNCH care practices																
(i) Family planning																
5. CPR among married or in union women	B	N	↑	↑	B	B	N	N	N	N	↓	↓	B	B	N	N
(ii) Maternal and newborn health																
6. Received any ANC from health facility	N	B	N	↑	B	B	N	N	B	N	N	N	N	N	↓	N
7. Received iron supplementation	N	N	N	N	B	B	N	N	B	N	N	N	N	N	N	N
8. Received TT2+	N	N	N	N	N	N	N	N	N	N	N	N	N	N	N	N
9. Took any birth preparedness measure	B	B	N	↑	B	B	N	↑	B	B	N	N	N	N	↓	N
10. Delivery at health facility	N	B	N	↑	B	B	N	↑	B	N	↑	N	B	N	N	N
11. Delivery assisted by skilled attendant	N	N	N	↑	B	B	N	↑	B	N	N	N	B	N	N	N
12. Received any PNC	B	N	↑	↑	B	N	N	N	B	N	N	N	B	N	N	N
13. Took thermal care of their newborn	N	N	N	N	B	N	N	N	B	B	↑	↑	N	N	N	N
14. Took clean cord care of their newborn	N	B	N	N	N	T	↓	↓	N	N	N	N	B	B	N	↑
15. Gave baby first milk (colostrums) to their newborn	B	B	N	N	B	N	N	N	B	N	N	N	N	B	N	N
(iii) Child health																
16. HHs contacted by HEWs for child health	B	N	N	N	N	N	N	N	N	N	N	N	N	N	N	N
17. Children with ARI received any treatment	N	N	N	N	N	N	N	N	N	N	N	N	N	N	N	N
18. Children with diarrhea received ORT	N	N	N	N	N	N	N	N	N	N	N	N	N	N	N	N
19. Children received all childhood vaccines	B	B	N	N	N	B	N	N	N	N	N	N	N	N	N	N

*N* Not statistically significant (*p* > 0.05), *B* bottom inequity present (*p* < 0.05), *T* top inequity present (*p* < 0.05); ↓ decline in bottom inequity (*p* < 0.05), ↑ increase in top inequity (*p* < 0.05)

Between the baseline and the follow-up surveys the age inequity (bottom) worsened for six of the 19 indicators; the education inequity worsened for three indicators and improved for two; the wealth inequity worsened for three indicators and improved for three; and the distance inequity worsened for two indicators and improved for one. Wald’s statistics and concentration indices corroborated for only six of the 20 statistically significant changes in inequities observed between the survey periods.

Interestingly top inequity was only observed for taking clean care of the umbilical cord of the newborn; the inequity was due to education.

## Discussion

This study examines the equity of four HEP outreach activity and 15 MNCH care practice indicators in the L10K areas according to women’s age, education, wealth, and distance from the nearest health facility, based on surveys conducted in December 2008 and December 2010, as well as changes in the inequities between the two survey periods. Inequities in the indicators of interest as of December 2010 were mainly due to age and education, followed by wealth and then distance from the nearest health facility. Although there were not many changes in the inequities in HEP outreach activity coverage and MNCH care practices between December 2008 and December 2010, the wealth inequity improved for three of the 19 indicators while it deteriorated for two of the indicators; the education inequity improved for two indicators and deteriorated for three indicators; the distance inequity improved for two indicators and declined for two indicators; while the age inequity deteriorated for six indicators.

L10K used the 30 by seven method mainly to minimize the cost of the survey field cost. However, the survey method is criticized because the interviewers may avoid hard-to-reach areas and non-responders are not revisited [21]. The hard-to-reach population and the non-responders are likely to have comparatively low MNCH care behavior and practices; as such, it is likely this study under-estimated inequity. Nevertheless, the observed changes in inequities reported here are most likely unbiased because the sampling method was consistent between the surveys; as such, the survey method bias was held constant when the changes in equity were assessed. Although the 30 by seven sampling method used non-probability sampling to select households for interviewing the target respondents raising the question regarding the accuracy of estimating the 95 % confidence intervals of the point estimates [30], a simulation exercise demonstrated that the accuracy of the variance estimates from such surveys are likely within the 95 % confidence limits [31].

The study was an adequacy design; as such, the observed changes in inequities could not be differentiated from secular trend from program influence (i.e., L10K or HEP or both). The exploratory analysis of this paper tested a large number of hypotheses; as such, some of the statistically significant findings may be spurious.

Two methods for measuring equity (i.e., Wald’s statistics and concentration index) were chosen for this study from the various methods available, which are described elsewhere [1, 26]. The concentration index value indicated whether the inequity was bottom or top; however, for Wald’s statistics the indicators according to the equity factor needed to be eye-balled to assess whether the inequity detected as statistically significant was top or bottom inequity. Only about half of the cross-sectional measures of statistically significant inequity during the follow-up survey corroborated between the two methods; while only six of the 20 statistically significant changes in equity measures observed between the baseline and follow-up surveys corroborated between the two methods. Since the different methods of measuring equity entail different sets of statistical assumptions, it would be advisable to use more than one method to measure equity and their changes over time.

The inequity in household visits by HEWs according to distance from the nearest health facility is of concern. However, it was reassuring to note that household visits by CHPs, model family households, and the possession of a family health card were not associated with distance from the nearest health facility, thus suggesting that the CHPs and model families are reaching the population in areas where HEW visits are relatively infrequent. It appears that maternal and newborn health care messages can reach areas where HEW visits are less frequent, as there were no inequities according to distance for 10 of the 15 maternal and newborn health inequity indicators analyzed in the follow-up survey. This finding is reinforced by the lack of inequity in possession of a family health card based on distance from a health facility.

Although there have been only two positive shifts in inequities in the indicators of interest according to distance from a health facility, it is encouraging to note that the proportion of the rural population who live more than one hour away from a health facility has been declining, from 22 % in December 2008 to just 9 % in December 2010. Thus, while those furthest from a health facility are still deprived of some aspects of MNCH care, they now represent a smaller fraction of the population.

Inequities in MNCH indicators according to women’s age existed for all four indicators of exposure to the HEP front-line workers. The L10K project supports the HEP in providing MNCH services. The expected pathway of the impact of L10K on MNCH behavior and practices is through the interactions of the HEP front-line workers with households. This equity analysis suggests that, to the extent that the impact of L10K occurs through HEW outreach activity, that impact would likely be inequitable according to women’s age, education and distance from a health facility, though not according to wealth. To the extent that L10K impacts MNCH behavior and practices through household visits by CHPs, then those impacts would likely be inequitable according to women’s age and education, but not by wealth or distance from a health facility.

This study has also shown significant improvements in the coverage of all the MNCH indicators other than that for treatment seeking behavior for ARI and the practice of ORT for managing diarrhea cases. It is understandable that there were no improvements in care seeking behavior for ARI because the provision for the service was not the part of HEP when the follow-up survey of this study was conducted. However, providing ORT had been within the HEP package of services. The HEP expects that there will be an improvement in the household care seeking behavior and practices for ARI and diarrhea case management following the expansion of the integrated community case management of common childhood illnesses in early 2011.

The existing strategies and policies of the HEP and L10K are less than optimum for minimizing inequities in MNCH services, whether according to women’s age, education, distance from a health facility, or wealth. The promotion of equitable MNCH services is essential if Ethiopia is to reach its MDG-related maternal and child health targets. Women age 15 to 19 and those who live more than an hour away from a health facility represent a small fraction of the target population for MNCH services (about 7 % and 9 %, respectively, during the follow-up survey). At the same time, the average distance from a health facility has been declining over time. Therefore, achieving equity of MNCH services by age and distance from a health facility would have only a modest (though still important) short-term impact on achieving the country’s overall health goals. For example, achieving equity in contraceptive use according to women’s age in L10K areas (i.e., enabling women age 15 to 19 to achieve the contraceptive prevalence rate exhibited by women age 20 to 34, shown in Additional file 1: Table S1) would mean an overall increase in contraceptive use of only 2 %, from 37 % (observed during the follow-up survey) to 39 %.

By contrast, the proportion of the women in rural L10K areas who have no education was substantial (78 %) in the follow-up survey. As such, addressing MNCH inequities according to women’s education will have greater impact and will significantly contribute toward achieving the country’s MDG targets related to maternal and child health. For example, achieving equity in MNCH indicators according to women’s education would mean an increase in the contraceptive prevalence rate from 37 % (during the follow-up survey) to 54 %. Similarly, institutional deliveries would increase from 11 % to 35 %, and deliveries assisted by health professionals would increase from 16 % to 41 %, among others.

The inequities in MNCH indicators according to education were most likely a combination of program uptake issues and differential targeting for MNCH services by the HEWs. Better-educated women may be more likely to proactively seek out and accept MNCH services provided by the HEP; as such, less educated women are lagging behind. Therefore, efforts to address inequity in the health sector should be complemented by other efforts in the broader social sector, where the introduction of strategies to reduce illiteracy and improve the population’s education will eventually eliminate education as a major source of inequity.

Assessing MNCH service equity due to religion and ethnicity was beyond the scope of this study. Nevertheless, there may be other health inequities related to these and other factors. Measuring such inequities is important for monitoring the effectiveness of existing policies for universal social services in Ethiopia. Accordingly, the equity of MNCH services by religion, ethnicity, region and place of residence should also be monitored using the EDHS.

One of the major assumptions for assessing equity of the HEP in providing MNCH services was that certain segments of the population (for instance, women less than 20 years old, women with no education, or women in the poorest health tercile) have greater health needs or less access to health services than others. However, this assumption was not tested. Future studies on equity of HEP services should test this assumption or assess equity according to health need.

## Conclusions

Inequities in the access and utilization of MNCH services due to women’s age and education, and due to household wealth and distance of the women’s household from the nearest health facility still exist in rural Ethiopia. The first step toward further promotion of equity in MNCH services in rural Ethiopia, so that the country can achieve and surpass its health-related MDGs, would be a stronger commitment by policy makers to addressing equity issues. Review of current strategies including the HEP will be required to ensure equity. Second, the entire health system must be made aware of the problem and the importance of addressing it in order to improve program performance. The third step would be to regularly monitor the equity of MNCH services by including equity indicators in the national Health Management Information System; this would be the cornerstone of a successful, ongoing strategy to address inequities in health care delivery and access.

## Additional file

Additional file 1: Table S1. Coverage and concentration indices for HEP outreach activities and MNCH care practice indicators by equity factors; and statistically significant Wald’s statistics for the inequities of those indicators during baseline (December 2008), follow-up (December 2010) periods and for the changes between the survey periods. (DOCX 111 kb)

## Abbreviations

ARI: Acute respiratory infection
CHP: Community health promoter
EDHS: Ethiopian demographic and health survey
HEP: Health extension program
HEW: Health extension worker
L10K: Last Ten Kilometers Project
MDG: Millennium Development Goal
MNCH: Maternal, newborn and child health
ORT: Oral rehydration therapy
