# Supplementary material for: Changes in equity of maternal, newborn, and child health care practices in 115 districts of rural Ethiopia: implications for the health extension program
Source: BMC Pregnancy Childbirth. 2015 Oct 5;15:238. doi: 10.1186/s12884-015-0668-z (PMC4595284; doi:10.1186/s12884-015-0668-z)
Supplement: Additional file 1: Table S1. — Coverage and concentration indices for HEP outreach activities and MNCH care practice indicators by equity factors; and statistically significant Wald’s statistics for the inequities of those indicators during baseline (December 2008), follow-up (December 2010) periods and for the changes between the survey periods. (DOCX 111 kb) [file 12884_2015_668_MOESM1_ESM.docx]

**Supplemental Table 1**: Coverage and concentration indices for HEP outreach activities and MNCH care practice indicators by equity factors; and statistically significant Wald’s statistics for the inequities of those indicators during baseline (December 2008), follow-up (December 2010) periods and for the changes between the survey periods

| **Equity factor** | | Baseline | | Follow-up | | Change | | Baseline | | Follow-up | | Change | |
| --- | --- | --- | --- | --- | --- | --- | --- | --- | --- | --- | --- | --- | --- |
|  |  | % | N | % | N | %-points |  | % | N | % | N | %-points |  |
|  |  | **HEP outreach activity coverage (among all respondents)** | | | | | | | | | | | |
|  |  | *Household visit by HEW* | | | | | | *Household visit by CHP* | | | | | |
| **Age group** | 15-19 | 27.9 | 274 | 39.1 | 271 | 11.3 | ** | 14.3 | 274 | 23.9 | 271 | 9.6 | ** |
|  | 20-34 | 36.4 | 2,810 | 45.2 | 2,796 | 8.9 | ** | 16.0 | 2,810 | 29.8 | 2,796 | 13.8 | ** |
|  | 35-49 | 40.7 | 803 | 56.1 | 844 | 15.5 | ** | 16.1 | 803 | 35.7 | 844 | 19.6 | ** |
|  | Concentration | 0.07 | ** | 0.074 | ** | 0.003 |  | 0.072 | ** | 0.085 | ** | 0.013 |  |
|  | Wald |  | ** |  | ** |  |  |  |  |  | ** |  |  |
| **Education** | None | 35.5 | 3,190 | 45.5 | 3,137 | 10 | ** | 14.5 | 3,190 | 29.8 | 3,137 | 15.3 | ** |
|  | Primary | 42.0 | 470 | 53.9 | 482 | 11.9 | ** | 20.6 | 470 | 34.9 | 482 | 14.3 | ** |
|  | Second/higher | 41.3 | 228 | 54.1 | 292 | 12.8 | ** | 26.3 | 228 | 33.3 | 292 | 6.9 | * |
|  | Concentration | 0.117 | ** | 0.094 | ** | -0.023 |  | 0.169 | ** | 0.100 | ** | -0.068 |  |
|  | Wald |  | ** |  | ** |  |  |  | ** |  |  |  | ** |
| **Wealth** | Poorest | 30.1 | 1,403 | 46.4 | 1,433 | 16.3 | ** | 18.1 | 1,403 | 34.8 | 1,433 | 16.7 | ** |
|  | Medium | 38.6 | 1,476 | 47.1 | 1,421 | 8.5 | ** | 14.1 | 1,476 | 28.4 | 1,421 | 14.2 | ** |
|  | Least poor | 42.8 | 1,009 | 48.3 | 1,057 | 5.5 |  | 15.5 | 1,009 | 28.2 | 1,057 | 12.7 | ** |
|  | Concentration | 0.093 | ** | 0.016 |  | -0.078 |  | -0.189 |  | -0.146 |  | 0.043 |  |
|  | Wald |  | ** |  |  |  | * |  |  |  | * |  |  |
| **Distance to** | <30 min. | 41.7 | 2,109 | 51.4 | 2,461 | 9.7 | ** | 15.8 | 2,109 | 30.5 | 2,461 | 14.7 | ** |
| **HF** | 30 min − <1 hr | 33.9 | 916 | 40.6 | 1,082 | 6.6 | * | 16.7 | 916 | 32.5 | 1,082 | 15.8 | ** |
|  | 1+ hour | 27.2 | 863 | 38.3 | 368 | 11.2 | ** | 15.4 | 863 | 27.0 | 368 | 11.5 | ** |
|  | Concentration | 0.308 | ** | 0.131 | ** | -0.176 |  | 0.016 |  | -0.041 |  | -0.057 |  |
|  | Wald | 0.52 | ** | 0.59 | ** | 1.12 |  | 0.97 |  | 0.84 |  | 0.87 |  |
| **All women** |  |  | 3,888 |  | 3,911 |  | ** |  | 3,888 |  | 3,911 |  | ** |
|  | | *Model family households* | | | | | | *Households with family health cards* | | | | | |
| **Age group** | 15-19 | 5.3 | 274 | 19.5 | 271 | 14.2 | ** | 4.2 | 274 | 23.5 | 271 | 19.3 | ** |
|  | 20-34 | 9.0 | 2,810 | 26.4 | 2,796 | 17.4 | ** | 4.8 | 2,810 | 32.6 | 2,796 | 27.7 | ** |
|  | 35-49 | 12.4 | 803 | 36.0 | 844 | 23.6 | ** | 7.1 | 803 | 34.6 | 844 | 27.5 | ** |
|  | Concentration | 0.177 | ** | 0.09 | ** | -0.086 |  | 0.105 | * | 0.066 | ** | -0.039 |  |
|  | Wald |  | ** |  | ** |  |  |  | * |  | ** |  |  |
| **Education** | None | 9.1 | 3,190 | 27.5 | 3,137 | 18.4 | ** | 4.6 | 3,190 | 30.1 | 3,137 | 25.5 | ** |
|  | Primary | 9.9 | 470 | 30.8 | 482 | 20.8 | ** | 7.1 | 470 | 42.7 | 482 | 35.6 | ** |
|  | Second/higher | 13.2 | 228 | 28.8 | 292 | 15.6 | ** | 10.9 | 228 | 39.9 | 292 | 29.1 | ** |
|  | Concentration | 0.305 |  | 0.158 | * | -0.146 |  | 0.258 | * | 0.14 | ** | -0.119 |  |
|  | Wald |  |  |  |  |  |  |  | ** |  | ** |  |  |
| **Wealth** | Poorest | 7.2 | 1,403 | 31.1 | 1,433 | 24 | ** | 6.9 | 1,403 | 30.3 | 1,433 | 23.4 | ** |
|  | Medium | 11.2 | 1,476 | 28.9 | 1,421 | 17.8 | ** | 3.9 | 1,476 | 31.9 | 1,421 | 27.9 | ** |
|  | Least poor | 10.0 | 1,009 | 22.5 | 1,057 | 12.5 | ** | 4.9 | 1,009 | 35.9 | 1,057 | 31.0 | ** |
|  | Concentration | 0.399 |  | -0.344 |  | -0.743 |  | 0.898 |  | 0.073 |  | -0.825 |  |
|  | Wald |  | ** |  |  |  | ** |  | ** |  |  |  | ** |
| **Distance to** | <30 min. | 9.5 | 2,109 | 29.2 | 2,461 | 19.7 | ** | 4.4 | 2,109 | 33.4 | 2,461 | 29 | ** |
| **HF** | 30 min − <1 hr | 11.7 | 916 | 27.4 | 1,082 | 15.7 | ** | 6.6 | 916 | 30.1 | 1,082 | 23.5 | ** |
|  | 1+ hour | 6.8 | 863 | 21.6 | 368 | 14.8 | ** | 5.9 | 863 | 32.1 | 368 | 26.2 | ** |
|  | Concentration | -0.195 |  | 0.023 |  | 0.218 |  | -0.158 |  | 0.005 |  | 0.163 |  |
|  | Wald |  |  |  |  |  |  |  |  |  |  |  |  |
| **All women** |  | 9.4 | 3,888 | 28.0 | 3,911 | 18.6 | ** | 5.3 | 3,888 | 32.4 | 3,911 | 27.1 | ** |
|  | | **MNCH care practices** | | | | | | | | | | | |
|  |  | ***Family planning (among women of reproductive age)*** | | | | | | | | | | | |
|  | | *Currently using any contraceptive method* | | | | | |  |  |  |  |  |  |
| **Age group** | 15-19 | 30.7 | 152 | 23.0 | 79 | -7.8 |  |  |  |  |  |  |  |
|  | 20-34 | 30.1 | 1,627 | 42.5 | 909 | 12.4 | ** |  |  |  |  |  |  |
|  | 35-49 | 21.6 | 538 | 39.3 | 376 | 17.6 | ** |  |  |  |  |  |  |
|  | Concentration | -0.055 | * | 0.014 |  | 0.069 | ** |  |  |  |  |  |  |
|  | Wald |  | ** |  | ** |  | ** |  |  |  |  |  |  |
| **Education** | None | 26.1 | 1,896 | 38.2 | 1,102 | 12.1 | ** |  |  |  |  |  |  |
|  | Primary | 33.3 | 289 | 47.0 | 162 | 13.7 | ** |  |  |  |  |  |  |
|  | Second/higher | 47.2 | 132 | 55.2 | 100 | 8 |  |  |  |  |  |  |  |
|  | Concentration | 0.02 |  | 0.069 | ** | 0.049 |  |  |  |  |  |  |  |
|  | Wald |  | ** |  | ** |  |  |  |  |  |  |  |  |
| **Wealth** | Poorest | 22.0 | 759 | 40.4 | 457 | 18.4 | ** |  |  |  |  |  |  |
|  | Medium | 28.1 | 927 | 41.5 | 495 | 13.4 | ** |  |  |  |  |  |  |
|  | Least poor | 35.8 | 631 | 39.4 | 413 | 3.6 |  |  |  |  |  |  |  |
|  | Concentration | 0.063 | ** | -0.015 |  | -0.078 | ** |  |  |  |  |  |  |
|  | Wald |  | ** |  |  |  | ** |  |  |  |  |  |  |
| **Distance to** | <30 min. | 30.6 | 1,255 | 43.3 | 905 | 12.7 | ** |  |  |  |  |  |  |
| **HF** | 30 min − <1 hr | 30.2 | 523 | 34.6 | 335 | 4.4 |  |  |  |  |  |  |  |
|  | 1+ hour | 20.5 | 539 | 35.3 | 125 | 14.8 | ** |  |  |  |  |  |  |
|  | Concentration | 0.052 | ** | 0.086 | ** | 0.034 |  |  |  |  |  |  |  |
|  | Wald |  | ** |  | ** |  |  |  |  |  |  |  |  |
| **All women** |  | 28.2 | 2,316 | 40.5 | 1,365 | 12.3 | ** |  |  |  |  |  |  |
|  | | ***Maternal & newborn health (among women with children 0 to 11 months)*** | | | | | | | | | | | |
|  | | *Received ANC during last pregnancy* | | | | | | *Received TT2+ during last pregnancy* | | | | | |
| **Age group** | 15-19 | 50.1 | 128 | 60.3 | 127 | 10.2 |  | 33.7 | 128 | 32.5 | 127 | -1.1 |  |
|  | 20-34 | 50.8 | 1,179 | 66.0 | 1,184 | 15.2 | ** | 41.8 | 1,179 | 42.9 | 1,184 | 1.1 |  |
|  | 35-49 | 50.9 | 218 | 72.0 | 261 | 21.1 | ** | 37.1 | 218 | 45.2 | 261 | 8.1 |  |
|  | Concentration | -0.009 |  | 0.038 | ** | 0.048 | ** | 0.005 |  | 0.025 |  | 0.020 |  |
|  | Wald |  |  |  | * |  |  |  |  |  |  |  |  |
| **Education** | None | 46.7 | 1,223 | 63.1 | 1,239 | 16.4 | ** | 39.4 | 1,223 | 40.6 | 1,239 | 1.2 |  |
|  | Primary | 62.2 | 205 | 76.0 | 205 | 13.8 | ** | 42.9 | 205 | 48.4 | 205 | 5.5 |  |
|  | Second/higher | 78.4 | 96 | 85.0 | 128 | 6.6 |  | 48.6 | 96 | 50.4 | 128 | 1.8 |  |
|  | Concentration | 0.110 | ** | 0.096 | ** | -0.013 |  | 0.053 | ** | 0.061 |  | 0.008 |  |
|  | Wald |  | ** |  | ** |  |  |  |  |  | * |  |  |
| **Wealth** | Poorest | 43.0 | 557 | 62.5 | 562 | 19.6 | ** | 37.0 | 557 | 36.3 | 562 | -0.6 |  |
|  | Medium | 50.3 | 553 | 63.9 | 579 | 13.6 | ** | 39.9 | 553 | 44.6 | 579 | 4.6 |  |
|  | Least poor | 62.0 | 415 | 75.3 | 431 | 13.4 | ** | 45.7 | 415 | 47.5 | 431 | 1.8 |  |
|  | Concentration | 0.081 | ** | 0.042 |  | -0.039 |  | 0.052 | ** | 0.062 | * | 0.010 |  |
|  | Wald |  | ** |  | ** |  |  |  |  |  | * |  |  |
| **Distance to** | <30 min. | 51.3 | 828 | 68.6 | 978 | 17.3 | ** | 44.5 | 828 | 44.6 | 978 | 0.1 |  |
| **HF** | 30 min − <1 hr | 60.0 | 338 | 63.8 | 445 | 3.8 |  | 38.7 | 338 | 39.0 | 445 | 0.3 |  |
|  | 1+ hour | 40.8 | 358 | 61.1 | 149 | 20.3 | ** | 32.5 | 358 | 38.6 | 149 | 6.1 |  |
|  | Concentration | 0.045 |  | 0.043 | * | -0.003 |  | 0.096 | ** | 0.042 |  | -0.054 |  |
|  | Wald |  | ** |  |  |  | ** |  | ** |  |  |  |  |
| **All women** |  | 50.8 | 1,524 | 66.5 | 1,572 | 15.8 | ** | 40.4 | 1,524 | 42.4 | 1,572 | 2.0 |  |
|  | | *Received iron supplementation* | | | | | | *Took any birth preparedness measure* | | | | | |
| **Age group** | 15-19 | 11.6 | 128 | 25.0 | 127 | 13.4 | ** | 65.2 | 128 | 64.1 | 127 | -1.1 |  |
|  | 20-34 | 10.7 | 1,179 | 27.3 | 1,184 | 16.6 | ** | 69.1 | 1,179 | 75.2 | 1,184 | 6.0 | ** |
|  | 35-49 | 8.1 | 218 | 31.2 | 261 | 23.1 | ** | 68.7 | 218 | 74.1 | 261 | 5.3 |  |
|  | Concentration | 0.039 |  | 0.046 | * | 0.007 |  | 0.005 |  | 0.035 | ** | 0.029 | ** |
|  | Wald |  |  |  |  |  |  |  |  |  | ** |  |  |
| **Education** | None | 8.9 | 1,223 | 25.1 | 1,239 | 16.2 | ** | 66.6 | 1,223 | 70.8 | 1,239 | 4.2 |  |
|  | Primary | 12.6 | 205 | 34.8 | 205 | 22.2 | ** | 72.7 | 205 | 84.3 | 205 | 11.7 | ** |
|  | Second/higher | 24.8 | 96 | 42.2 | 128 | 17.4 | ** | 87.2 | 96 | 89.2 | 128 | 2.0 |  |
|  | Concentration | 0.165 | ** | 0.138 | ** | -0.027 |  | 0.044 | ** | 0.087 | ** | 0.043 | ** |
|  | Wald |  | ** |  | ** |  |  |  | ** |  | ** |  |  |
| **Wealth** | Poorest | 9.4 | 557 | 28.3 | 562 | 19.0 | ** | 61.9 | 557 | 69.2 | 562 | 7.3 | ** |
|  | Medium | 10.1 | 553 | 23.5 | 579 | 13.4 | ** | 71.2 | 553 | 75.1 | 579 | 3.9 |  |
|  | Least poor | 12.2 | 415 | 32.7 | 431 | 20.5 | ** | 74.7 | 415 | 79.1 | 431 | 4.4 |  |
|  | Concentration | 0.064 |  | 0.004 |  | -0.061 |  | 0.044 | ** | 0.038 | ** | -0.007 |  |
|  | Wald |  |  |  | ** |  |  |  | ** |  | ** |  |  |
| **Distance to** | <30 min. | 10 | 828 | 29.0 | 978 | 19.1 | ** | 70.2 | 828 | 74.3 | 978 | 4.1 |  |
| **HF** | 30 min − <1 hr | 13.6 | 338 | 26.6 | 445 | 13.0 | ** | 69.6 | 338 | 71.2 | 445 | 1.6 |  |
|  | 1+ hour | 8.4 | 358 | 22.8 | 149 | 14.4 | ** | 64.6 | 358 | 81.2 | 149 | 16.6 | ** |
|  | Concentration | 0.028 |  | 0.065 |  | 0.038 |  | 0.009 |  | 0.003 |  | -0.006 |  |
|  | Wald |  |  |  |  |  |  |  |  |  |  |  | ** |
| **All women** |  | 10.4 | 1,524 | 27.8 | 1,572 | 17.3 | ** | 68.8 | 1,524 | 74.1 | 1,572 | 5.4 | ** |
|  | | *Last delivery at a health facility* | | | | | | *Last delivery attended by skilled birth attendant* | | | | | |
| **Age group** | 15-19 | 8.4 | 128 | 13.6 | 127 | 5.2 |  | 12.3 | 128 | 15.2 | 127 | 2.8 |  |
|  | 20-34 | 4.8 | 1,179 | 9.9 | 1,184 | 5.1 | ** | 9.3 | 1,179 | 14.6 | 1,184 | 5.3 | ** |
|  | 35-49 | 5.6 | 218 | 12.4 | 261 | 6.8 | ** | 9.1 | 218 | 14.5 | 261 | 5.4 | * |
|  | Concentration | -0.137 |  | 0.104 | ** | 0.242 | ** | -0.074 |  | 0.043 |  | 0.117 | ** |
|  | Wald |  |  |  |  |  |  |  |  |  |  |  |  |
| **Education** | None | 4.4 | 1,223 | 7.0 | 1,239 | 2.6 | ** | 8.1 | 1,223 | 10.6 | 1,239 | 2.5 | ** |
|  | Primary | 5.1 | 205 | 15.0 | 205 | 9.8 | ** | 10.3 | 205 | 20.0 | 205 | 9.7 | ** |
|  | Second/higher | 15.8 | 96 | 38.6 | 128 | 22.8 | ** | 25.9 | 96 | 44.9 | 128 | 19.0 | ** |
|  | Concentration | 0.14 | ** | 0.33 | ** | 0.19 | * | 0.11 | ** | 0.235 | ** | 0.125 | ** |
|  | Wald |  | ** |  | ** |  |  |  | ** |  | ** |  |  |
| **Wealth** | Poorest | 3.4 | 557 | 9.6 | 562 | 6.1 | ** | 7.5 | 557 | 13.1 | 562 | 5.6 | ** |
|  | Medium | 5.2 | 553 | 6.9 | 579 | 1.7 |  | 9.2 | 553 | 11.5 | 579 | 2.3 |  |
|  | Least poor | 7.7 | 415 | 17.0 | 431 | 9.3 | ** | 12.6 | 415 | 20.8 | 431 | 8.2 | ** |
|  | Concentration | 0.11 | * | 0.532 |  | 0.422 |  | 0.045 |  | 0.092 |  | 0.047 |  |
|  | Wald |  | * |  | ** |  | * |  |  |  | ** |  |  |
| **Distance to** | <30 min. | 6.0 | 828 | 12.3 | 978 | 6.3 | ** | 12.0 | 828 | 16.9 | 978 | 4.9 | ** |
| **HF** | 30 min − <1 hr | 6.0 | 338 | 8.1 | 445 | 2.1 |  | 7.9 | 338 | 11.1 | 445 | 3.2 |  |
|  | 1+ hour | 2.7 | 358 | 7.2 | 149 | 4.5 | ** | 5.2 | 358 | 10.4 | 149 | 5.1 |  |
|  | Concentration | 0.128 |  | 0.695 |  | 0.567 |  | 0.149 | ** | 0.156 |  | 0.007 |  |
|  | Wald |  | * |  | ** |  |  |  | ** |  | ** |  |  |
| **All women** |  | 5.2 | 1,524 | 10.6 | 1,572 | 5.4 | ** | 9.5 | 1,524 | 14.6 | 1,572 | 5.1 | ** |
|  | | *Received any PNC after last childbirth* | | | | | | *Took thermal care of the newborn* | | | | | |
| **Age group** | 15-19 | 4.3 | 128 | 8.4 | 127 | 4.1 |  | 13.1 | 128 | 17.5 | 127 | 4.4 |  |
|  | 20-34 | 4.6 | 1,179 | 13.7 | 1,184 | 9.1 | ** | 12.1 | 1,179 | 24.1 | 1,184 | 12.0 | ** |
|  | 35-49 | 2.4 | 218 | 18.3 | 261 | 15.9 | ** | 8.0 | 218 | 21.2 | 261 | 13.2 | ** |
|  | Concentration | -0.038 |  | 0.164 |  | 0.201 | ** | -0.052 |  | 0.005 |  | 0.058 |  |
|  | Wald |  |  |  | ** |  | * |  |  |  |  |  |  |
| **Education** | None | 3.4 | 1,223 | 12.4 | 1,239 | 9 | ** | 11.9 | 1,223 | 21.5 | 1,239 | 9.6 | ** |
|  | Primary | 9.1 | 205 | 20.9 | 205 | 11.7 | ** | 9.3 | 205 | 23.6 | 205 | 14.2 | ** |
|  | Second/higher | 4.8 | 96 | 18.8 | 128 | 14 | ** | 12.9 | 96 | 37.9 | 128 | 25.0 | ** |
|  | Concentration | 0.13 | ** | .977 |  | 0.964 |  | 0.011 |  | 0.071 |  | 0.06 |  |
|  | Wald |  | ** |  | ** |  |  |  |  |  | ** |  |  |
| **Wealth** | Poorest | 3.8 | 557 | 9.9 | 562 | 6 | ** | 15.1 | 557 | 17.4 | 562 | 2.4 |  |
|  | Medium | 4.1 | 553 | 15.7 | 579 | 11.6 | ** | 10.0 | 553 | 23.4 | 579 | 13.4 | ** |
|  | Least poor | 5.0 | 415 | 17.2 | 431 | 12.2 | ** | 9.0 | 415 | 30.2 | 431 | 21.2 | ** |
|  | Concentration | 0.007 |  | 0.784 |  | 0.777 |  | -0.062 | * | 0.128 | ** | 0.19 | ** |
|  | Wald |  |  |  | ** |  |  |  | * |  | ** |  | ** |
| **Distance to** | <30 min. | 4.4 | 828 | 16.5 | 978 | 12.1 | ** | 12.2 | 828 | 24.2 | 978 | 12 | ** |
| **HF** | 30 min − <1 hr | 2.6 | 338 | 10.0 | 445 | 7.4 | ** | 10.7 | 338 | 22.3 | 445 | 11.6 | ** |
|  | 1+ hour | 5.5 | 358 | 10.2 | 149 | 4.7 | * | 11.2 | 358 | 18.5 | 149 | 7.4 | * |
|  | Concentration | 0.033 |  | -0.454 |  | -0.487 |  | 0.022 |  | 0.026 |  | 0.004 |  |
|  | Wald |  |  |  | ** |  |  |  |  |  |  |  |  |
| **All women** |  | 4.2 | 1,524 | 14.0 | 1,572 | 9.8 | ** | 11.6 | 1,524 | 23.1 | 1,572 | 11.5 | ** |
|  | | *Took clean care of umbilical cord* | | | | | | *Gave baby colostrums (first milk)* | | | | | |
| **Age group** | 15-19 | 30.8 | 128 | 29.0 | 127 | -1.8 |  | 46.9 | 128 | 48.0 | 127 | 1.0 |  |
|  | 20-34 | 30.9 | 1,179 | 39.2 | 1,184 | 8.4 | ** | 42.7 | 1,179 | 50.0 | 1,184 | 7.3 | ** |
|  | 35-49 | 32.9 | 218 | 38.9 | 261 | 6.0 |  | 53.7 | 218 | 59.2 | 261 | 5.5 |  |
|  | Concentration | 0.011 |  | 0.049 | ** | 0.039 |  | 0.053 | ** | 0.032 | ** | -0.021 |  |
|  | Wald |  |  |  | * |  |  |  | ** |  | ** |  |  |
| **Education** | None | 30.1 | 1,223 | 39.2 | 1,239 | 9.1 | ** | 44.3 | 1,223 | 50.5 | 1,239 | 6.2 | ** |
|  | Primary | 31.3 | 205 | 36.5 | 205 | 5.2 |  | 44.7 | 205 | 48.2 | 205 | 3.5 |  |
|  | Second/higher | 43.3 | 96 | 33.2 | 128 | -10.1 |  | 49.2 | 96 | 65.3 | 128 | 16.1 | ** |
|  | Concentration | 0.05 |  | -0.107 | ** | -0.157 | ** | -0.07 |  | 0.032 |  | 0.103 |  |
|  | Wald |  | ** |  |  |  | * |  |  |  | ** |  |  |
| **Wealth** | Poorest | 27.7 | 557 | 34.9 | 562 | 7.2 |  | 42.2 | 557 | 50.1 | 562 | 8.0 | * |
|  | Medium | 28.3 | 553 | 37.7 | 579 | 9.4 | ** | 44.4 | 553 | 47.1 | 579 | 2.7 |  |
|  | Least poor | 39.6 | 415 | 43.9 | 431 | 4.3 |  | 48.3 | 415 | 58.9 | 431 | 10.6 | ** |
|  | Concentration | 0.096 | ** | 0.063 | * | -0.033 |  | 0.072 | ** | 0.035 |  | -0.037 |  |
|  | Wald |  | ** |  |  |  |  |  |  |  | ** |  |  |
| **Distance to** | <30 min. | 31.2 | 828 | 42.7 | 978 | 11.6 | ** | 45.2 | 828 | 48.7 | 978 | 3.5 |  |
| **HF** | 30 min − <1 hr | 29.0 | 338 | 30.6 | 445 | 1.6 |  | 44.9 | 338 | 55.2 | 445 | 10.3 | ** |
|  | 1+ hour | 33.1 | 358 | 32.7 | 149 | -0.3 |  | 43.2 | 358 | 57.8 | 149 | 14.6 | ** |
|  | Concentration | -0.018 |  | 0.12 | ** | 0.138 | ** | -0.026 |  | -0.059 | ** | -0.033 |  |
|  | Wald |  |  |  | ** |  |  |  |  |  |  |  |  |
| **All women** |  | 31.1 | 1,524 | 38.4 | 1,572 | 7.2 | ** | 44.6 | 1,524 | 51.4 | 1,572 | 6.8 | ** |
|  | | **Child health (among children 0 to 23 months)** | | | | | | | | | | | |
|  | | *Households contacted by HEWs for child health* | | | | | | *Children with ARI received any treatment* | | | | | |
| **Age group** | 15-19 | 7.4 | 196 | 16.1 | 193 | 8.7 | ** | 48.4 | 196 | 37.9 | 193 | -10.6 |  |
|  | 20-34 | 11.3 | 2,138 | 23.0 | 2,373 | 11.7 | ** | 30.7 | 2,138 | 36.0 | 2,373 | 5.3 |  |
|  | 35-49 | 11.6 | 459 | 30.6 | 563 | 19.0 | ** | 29.0 | 459 | 31.2 | 563 | 2.2 |  |
|  | Concentration | 0.13 | ** | 0.056 | * | -0.073 |  | -0.076 |  | -0.056 |  | 0.020 |  |
|  | Wald |  |  |  | ** |  |  |  |  |  |  |  |  |
| **Education** | None | 10.5 | 2,281 | 23.3 | 2,515 | 12.8 | ** | 29.6 | 2,281 | 33.8 | 2,515 | 4.2 |  |
|  | Primary | 11.4 | 344 | 26.0 | 383 | 14.6 | ** | 46.0 | 344 | 40.1 | 383 | -5.9 |  |
|  | Second/higher | 17.8 | 168 | 27.5 | 232 | 9.7 | ** | 24.0 | 168 | 44.8 | 232 | 20.8 |  |
|  | Concentration | 0.217 |  | 0.120 | * | -0.097 |  | 0.014 |  | 0.01 |  | -0.004 |  |
|  | Wald |  | ** |  |  |  |  |  | * |  |  |  |  |
| **Wealth** | Poorest | 10.8 | 1,004 | 20.3 | 1,137 | 9.5 | ** | 28.2 | 1,004 | 27.1 | 1,137 | -1.1 |  |
|  | Medium | 10.6 | 1,040 | 26.7 | 1,150 | 16.1 | ** | 31.9 | 1,040 | 41.5 | 1,150 | 9.6 |  |
|  | Least poor | 11.9 | 749 | 24.9 | 842 | 13.0 | ** | 33.9 | 749 | 37.1 | 842 | 3.2 |  |
|  | Concentration | -0.064 |  | 0.100 | * | 0.164 |  | 0.027 |  | 0.053 |  | 0.026 |  |
|  | Wald |  |  |  |  |  |  |  |  |  |  |  |  |
| **Distance to** | <30 min. | 11.5 | 1,506 | 25.2 | 1,927 | 13.8 | ** | 30.9 | 1,506 | 38.7 | 1,927 | 7.8 |  |
| **HF** | 30 min − <1 hr | 11.3 | 643 | 21.7 | 891 | 10.5 | ** | 28.9 | 643 | 34.8 | 891 | 5.9 |  |
|  | 1+ hour | 9.8 | 644 | 21.8 | 311 | 12.1 | ** | 33.6 | 644 | 18.8 | 311 | -14.8 |  |
|  | Concentration | 0.375 |  | 0.106 |  | -0.269 |  | 0.009 |  | 0.012 |  | 0.003 |  |
|  | Wald |  |  |  |  |  |  |  |  |  |  |  |  |
| **All women** |  | 11.0 | 2,793 | 23.9 | 3,129 | 12.9 | ** | 31.3 | 2,793 | 35.3 | 3,129 | 4.0 |  |
|  | | *Children with diarrhea received ORT* | | | | | | *Children aged 12 to 23 months received all vaccines* | | | | | |
| **Age group** | 15-19 | 42.0 | 196 | 54.7 | 193 | 12.7 |  | 36.7 | 68 | 42.3 | 66 | 5.7 |  |
|  | 20-34 | 43.9 | 2,138 | 47.4 | 2,373 | 3.5 |  | 44.7 | 959 | 49.3 | 1,189 | 4.6 |  |
|  | 35-49 | 44.2 | 459 | 51.2 | 563 | 7.0 |  | 45.0 | 242 | 58.3 | 302 | 13.3 | ** |
|  | Concentration | 0.058 |  | 0.010 |  | -0.048 |  | 0.021 |  | 0.052 | ** | 0.031 |  |
|  | Wald |  |  |  |  |  |  |  |  |  | ** |  |  |
| **Education** | None | 41.4 | 2,281 | 47.9 | 2,515 | 6.5 |  | 42.5 | 1,058 | 49.4 | 1,276 | 6.8 | * |
|  | Primary | 54.1 | 344 | 49.4 | 383 | -4.7 |  | 50.5 | 138 | 55.2 | 178 | 4.7 |  |
|  | Second/higher | 51.9 | 168 | 50.9 | 232 | -1.1 |  | 59.0 | 72 | 60.7 | 103 | 1.7 |  |
|  | Concentration | 0.143 | * | -0.001 |  | -0.143 |  | 0.055 |  | 0.106 | ** | 0.051 |  |
|  | Wald |  |  |  |  |  |  |  | ** |  |  |  |  |
| **Wealth** | Poorest | 42.7 | 1,004 | 46.1 | 1,137 | 3.4 |  | 45.5 | 447 | 49.0 | 574 | 3.5 |  |
|  | Medium | 46.9 | 1,040 | 45.6 | 1,150 | -1.4 |  | 41.6 | 487 | 48.5 | 571 | 6.8 |  |
|  | Least poor | 40.9 | 749 | 54.4 | 842 | 13.6 | ** | 46.7 | 335 | 56.5 | 412 | 9.8 | ** |
|  | Concentration | 0.012 |  | 0.06 |  | 0.048 |  | -0.023 |  | 0.043 |  | 0.066 |  |
|  | Wald |  |  |  |  |  |  |  |  |  | * |  |  |
| **Distance to** | <30 min. | 41.7 | 1,506 | 49.4 | 1,927 | 7.7 |  | 44.3 | 678 | 49.7 | 949 | 5.4 |  |
| **HF** | 30 min − <1 hr | 44.7 | 643 | 45.4 | 891 | 0.7 |  | 48.2 | 305 | 51.5 | 446 | 3.3 |  |
|  | 1+ hour | 47.6 | 644 | 49.8 | 311 | 2.2 |  | 40.4 | 286 | 55.5 | 162 | 15.1 | ** |
|  | Concentration | -0.053 |  | 0.006 |  | 0.059 |  | -0.015 |  | -0.017 |  | -0.002 |  |
|  | Wald |  |  |  |  |  |  |  |  |  |  |  |  |
| **All women** |  | 43.8 | 2,793 | 48.3 | 3,129 | 4.6 |  | 44.3 | 1,269 | 50.8 | 1,557 | 6.4 | ** |
